# High‐Performance X‐Ray Imaging using Lanthanide Metal–Organic Frameworks

**DOI:** 10.1002/advs.202207004

**Published:** 2023-03-22

**Authors:** Xintong Zhang, Haiyi Qiu, Wang Luo, Kaofeng Huang, Ying Chen, Jiacheng Zhang, Bohan Wang, Daoling Peng, Yu Wang, Kezhi Zheng

**Affiliations:** ^1^ Guangdong Provincial Key Laboratory of Quantum Engineering and Quantum Material School of Physics and Telecommunication Engineering South China Normal University Guangzhou 510006 China; ^2^ SZU–NUS Collaborative Innovation Center ICL 2DMOST Institute of Microscale Optoelectronics Shenzhen University Shenzhen 518060 China; ^3^ Key Laboratory of Theoretical Chemistry of Environment Ministry of Education School of Environment South China Normal University Guangzhou 510006 China

**Keywords:** metal–organic framework, radiography, radioluminescence, scintillators, triplet energy transfer

## Abstract

Scintillating materials that convert ionizing radiation into low‐energy photons hold great potential for radiation detection, nondestructive inspection, medical radiography, and space exploration. However, organic scintillators are characterized by low radioluminescence, while bulky inorganic scintillators are not suitable for the development of flexible detectors. Here, high‐resolution X‐ray imaging using solution‐processable lanthanide‐based metal–organic frameworks as microscale scintillators is demonstrated. Mechanistic studies suggest that lanthanide ions absorb X‐rays to generate high‐density molecular triplet excitons, and excited linkers subsequently sensitize lanthanide ions via nonradiative resonance energy transfer. Furthermore, the crystalline nature offers a delocalized electronic feature rather than isolated subunits, which enables direct trapping of charge carriers by lanthanide emitters. By controlling the concentration ratio between Tb^3+^ and Eu^3+^ ions, efficient and color‐tunable radioluminescence of lanthanide ions can be achieved. When coupled with elastic, transparent polymer matrices, these metal–organic framework‐based microscintillators allow the fabrication of flexible X‐ray detectors. Such detectors feature a detection limit of 23 nGy s^−1^, which is 240 times lower than the typical radiation dose for medical diagnosis. X‐ray imaging with resolution higher than 16.6 line pairs per millimeter is further demonstrated. These findings provide insight into the future design of hybrid scintillators for optoelectronics and X‐ray sensing and imaging.

## Graphical abstract

Here, a novel class of lanthanide metal–organic‐framework‐based X‐ray scintillators with high photostability and efficient radioluminescnce is reported. By coupling with elastic, transparent polymer matrices, these metal–organic‐framework‐based microphosphors allow the fabrication of flexible X‐ray detectors with low detection limit, providing inspirations for high‐sensitivity X‐ray sensing and high‐resolution X‐ray imaging.

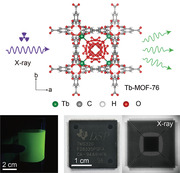

## 1 Introduction

Scintillators are radiation‐responsive materials that can convert high‐energy X‐rays or gamma‐rays into visible or near‐visible photons.^[^ ^1^ ^−^ ^6^ ^]^ Compared with organic scintillators comprising light elements,^[^ ^7^ ^−^ ^9^ ^]^ inorganic scintillators with heavy atomic constituents have been extensively used in a wide range of radiation detection applications,^[^ ^10^ ^−^ ^14^ ^]^ mainly due to their strong X‐ray absorption, large attenuation coefficient, and high optical stability.^[^ ^15^ ^−^ ^17^ ^]^ Despite being commercially available, these inorganic scintillators are grown by the high‐temperature growth method and generally form single crystals or polycrystalline ceramics, making them unsuitable for fabrication of flexible X‐ray detectors.^[^ ^18^ ^−^ ^21^ ^]^ Considerable efforts have been made to prepare solution processable nanoscintillators at low temperature. Of them, all‐inorganic lead‐based perovskite nanocrystals have been considered an emerging class of nanoscintillators with attractive attributes, including high‐emission quantum yield, fast scintillation response, and low detection limit.^[^ ^2^, ^22^ ^−^ ^24^ ^]^ However, perovskite‐based nanoscintillators suffer from irreversible chemical degradation even under ambient conditions, which severely limit their practical uses. In addition, the toxicity of elemental lead poses persistent, acute health, and environmental concerns.

Organic–inorganic hybrids generally inherit optical characteristics from their parent components.^[^ ^25^ ^−^ ^28^ ^]^ In particular, metal–organic frameworks (MOFs) offer great flexibility in optical tuning through simple manipulation of organic and inorganic building blocks. Additionally, MOFs have been shown to be highly resistant to optical irradiation, heat, and moisture. Recently, high‐*Z* fluorescent MOF nanocrystals have been developed as a new class of scintillators for radiation detection and X‐ray imaging.^[^ ^29^, ^30^ ^]^ The high‐intensity radioluminescence from organic emitters was attributed to the enhanced X‐ray absorption due to the incorporation of heavy metal atoms. Given the poor photostability of organic emitters, these MOF nanoscintillators usually suffer from photoblinking and bleaching.

**Figure 1:**
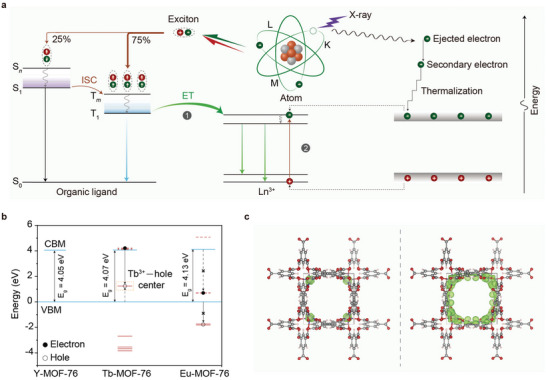
X‐ray‐excited radioluminescence in lanthanide metal–organic frameworks. a) Mechanistic diagram of the scintillation process in Ln‐MOFs. Upon X‐ray irradiation, high‐energy photoelectrons are ejected mainly from the inner shells of heavy metal atoms in the Ln‐MOF matrix due to the photoelectric effect. Electrons in the outer shells subsequently occupy the inner‐shell voids and generate low‐energy secondary photons, which further interact with the MOFs. The secondary photons excite organic ligands and produce singlet and triplet excitons in a ratio of 1:3. The excitation energy is transferred from the long‐lived triplet state of the molecules to their adjacent lanthanide ions, resulting in lanthanide‐activated radioluminescence (process 1). Concurrently, the secondary photons excite the Ln‐MOF lattice and generate free charge carriers that could be trapped by lanthanide ions for radioluminescence (process 2). ISC, intersystem crossing; ET, energy transfer; S, singlet; T, triplet. b) Calculated energies of the lanthanide 4f orbitals (in red) with respect to the host bands (in blue). Solid and dotted lines represent occupied and empty 4f orbitals, respectively. VBM, valence band maximum; CBM, conduction band minimum. c) Calculated partial charge densities (in green) of the VBM (left) and CBM (right) of Tb‐MOF‐76 microcrystals. Gray, red, and pink spheres denote C, O, and H atoms, respectively. Note that Tb atoms are blocked by the isosurface plot of charge density.

By virtue of highly stable optical transitions of metal ions, we reason that metal–organic frameworks comprising heavy lanthanide ions (Ln‐MOFs) should possess great potential for X‐ray conversion to low energy photons. Specifically, the heavy lanthanides can efficiently absorb X‐ray energy and generate secondary photons mainly through the photoelectric effect. In inelastic scattering, the redistribution of thermalized electron−hole pairs generates 75% triplet and 25% singlet excitons in organic molecules.^[^ ^9^, ^31^, ^32^ ^]^ Given the strong coupling and high degree of spectral overlap between molecules and lanthanides, lanthanide emitters not only facilitate intersystem crossing from molecular singlet to triplet states with high efficiency but also receive triplet‐state energy through nonradiative resonance energy transfer.^[^ ^33^, ^34^ ^]^ In this regard, Ln‐MOFs should allow sensitization of lanthanide emitters by utilizing high‐density triplet excitons, enabling intense radioluminescence (**Figure** **1**a, process 1).

Owing to the crystalline nature of molecular‐building blocks, lanthanide ions can be considered dopants in metal–organic frameworks, which bear a strong resemblance to lanthanide‐doped inorganic phosphors.^[^ ^35^ ^]^ Apart from receiving energy from molecular triplet excitons, lanthanide ions can be excited by trapping X‐ray‐generated charge carriers, followed by electron−hole radiative recombination (Figure 1a, process 2).

We chose 1,3,5‐benzenetricarboxylate (H_3_BTC) as the molecular‐building block to construct lanthanide‐based MOFs (Ln‐MOF‐76; Ln = Tb or Eu) with high rigidity and thermal stability. Moreover, the triplet state of H_3_BTC is close to the emitting states of Tb^3+^/Eu^3+^. The calculated energy gaps between the lowest molecular triplet state and the lanthanide‐emitting states are 1.07 and 1.48 eV for Tb^3+^ and Eu^3+^, respectively, suggesting a higher energy transfer rate from H_3_BTC to Tb^3+^ than to Eu^3+^. To evaluate the trapping ability of lanthanide ions, we calculated their 4f orbital energy with respect to the valence and conduction band edges of MOFs (Figure 1b and Figure S1, Supporting Information). In the case of Tb‐MOF‐76, the valence band maximum is mainly composed of Tb 4f orbitals, suggesting a high probability of hole trapping (Figure 1c). Considering that the empty 4f orbitals resonate with the conduction band minimum, Tb^3+^ ions can trap electrons efficiently, resulting in radioluminescence through electron−hole recombination. In contrast, the occupied 4f orbitals of Eu are located deep in the valence band, indicative of inefficient hole trapping and low electron−hole recombination probability (Figures S1 and S2, Supporting Information). Considered together, we speculate that Tb‐MOF‐76 should have higher radioluminescence intensity than its Eu counterpart because of a higher degree of spectral overlap and a higher probability of hole trapping at Tb sites.

## 2 Results and Discussion

**Figure 2:**
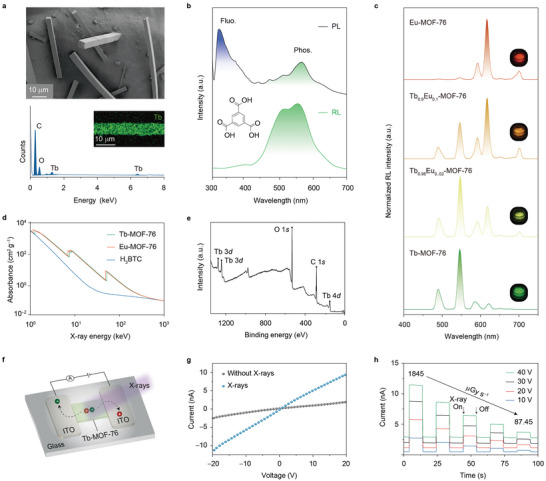
Photophysical investigations of X‐ray‐excited radioluminescence in lanthanide‐based MOF‐76 microscintillators. a) Scanning electron microscopy image (top panel) and energy‐dispersive X‐ray spectroscopy analysis (bottom panel) of Tb‐MOF‐76 microcrystals. The inset shows a uniform distribution of elemental Tb in a selected Tb‐MOF‐76 microcrystal. b) Normalized photoluminescence and radioluminescence spectra of the H_3_BTC molecules. Purple and green bands represent the fluorescence and phosphorescence of H_3_BTC molecules, respectively. Inset shows the atomic structure of an H_3_BTC molecule. c) Radioluminescence of MOF‐76 microcrystals doped with Tb^3+^ and Eu^3+^ activators at different concentrations. The dose rate of X‐ray irradiation was set to 1845 µGy s^−1^. Insets are photographs of the MOF‐76 microcrystals upon X‐ray irradiation. d) X‐ray absorption spectra of Tb‐MOF‐76, Eu‐MOF‐76, and H_3_BTC as a function of X‐ray energy. Attenuation coefficients were obtained from ref. [37]. e) X‐ray photoelectron spectroscopy of Tb‐MOF‐76 microcrystals. f) Schematic of the Ln‐MOF‐based photodetector. A layer of Tb‐MOF‐76 scintillators was spin‐coated onto the glass substrate for X‐ray photon‐carrier conversion and indium tin oxide (ITO) electrodes on the glass substrate are used for hole and electron extraction. The distance between the two ITO electrodes is 100 µm. g) *I*–*V* characteristics of the as‐prepared photodetector, measured with and without X‐rays at a dose rate of 1845 µGy s^−1^. h) Current responses of the fabricated photodetector, recorded under various bias voltages (from 40 to 10 V) and dose rates (from 1845 to 87.45 µGy s^−1^).

We synthesized Tb‐ and Eu‐MOFs at low temperatures (<100 °C).^[^ ^36^ ^]^ Fine‐tuning of crystal size and shape were achieved by controlling temperatures, types of lanthanide precursors, and reactant ratios (Figures S3−S5, Supporting Information). X‐ray diffraction (XRD) characterization shows that the patterns of samples under study can be easily indexed in accord with the simulated pattern of MOF‐76 (Figures S6−S8, Supporting Information). Tb‐MOF‐76 samples have the shape of a square prism with a length of up to several tens of micrometers, as manifested by scanning electron microscopy (SEM) images (**Figure** **2**a). Compositional analysis by energy‐dispersive X‐ray spectroscopy (EDX) confirms the presence of terbium dopants, and elemental mapping further reveals a uniform distribution of Tb elements in one single microparticle. Notably, these MOF microparticles possess high thermal stability. For instance, thermogravimetric analysis (TGA) shows a slight weight loss of Tb‐MOF‐76 microcrystals upon heating to 440 °C, which is ascribed to the desorption of water and DMF solvent molecules (Figure S9, Supporting Information).

We next investigated the X‐ray radioluminescence of the Ln‐MOFs under ambient conditions. The X‐ray‐enabled generation of triplet excitons was evidenced by comparing the photoluminescence and radioluminescence of H_3_BTC molecules alone (Figure 2b). When coupled with metal ions, both Tb‐ and Eu‐based MOFs show intense luminescence upon X‐ray irradiation, characterized by the signature line‐like emission of Tb^3+^ and Eu^3+^ (Figures S10−S12, Supporting Information). By comparison, the emission intensity of Tb^3+^ is much higher than that of Eu^3+^, corroborating the predicted efficient energy transfer and charge carrier trapping in Tb‐MOF‐76 systems (Figure S13, Supporting Information). By taking advantage of energy transfer between Tb^3+^ and Eu^3+^, emission color tuning was achieved by simple codoping of lanthanides. With increasing doping ratio of Eu^3+^ to Tb^3+^, emission color changed from red to green (Figure 2c, Figures S14–S15, and Table S1, Supporting Information).

As one of the critical performance indicators, the absorption coefficients of the Ln‐MOFs (Tb‐MOF‐76, *Z*_max_ = 65, K*α* = 52.0 keV; Εu‐MOF‐76, *Z*_max_ = 63, K*α* = 48.5 keV) as a function of X‐ray photon energy were compared with those of H_3_BTC ligands (*Z*_max_ = 8, K*α* = 0.525 keV) (Figure 2d). It is clear that high‐atomic‐number (high‐*Z*) rare‐earth elements are essential for large X‐ray absorption and efficient X‐ray scintillation. The underlying rationale is that the attenuation coefficient scales with the fourth power of the effective atomic number *Z*_eff_.^[^ ^20^ ^]^ Given the strong radioluminescence of Tb‐MOF‐76, we selected it as a model system to further investigate its optical response to X‐rays. X‐ray photoelectron spectroscopic (XPS) analysis was performed to unravel the kinetics of electrons escaping from the surface of Tb‐MOF‐76 microcrystals (Figure 2e). Photoemission peaks from Tb 3*d*, O 1*s*, C 1*s*, and Tb 4*d* confirm the material's excellent X‐ray absorption.

To shed more light on the photogeneration of charge carriers upon X‐ray irradiation, we fabricated a photoconductor using Tb‐MOF‐76 microcrystals as photoconductive material and measured the corresponding currents (Figure 2f). The generation of electrons and holes was confirmed by the substantial photoconductive gain (Figure 2g and Figure S16, Supporting Information). Notably, the Tb‐MOF‐based photoconductor shows repeatable, systematic photocurrent variation at different voltage biases (40−10 V) upon pulsed X‐ray irradiation with a dose rate varying from 1845 to 87.45 *µ*Gy s^−1^, recorded at a time interval of 10 s (Figure 2h and Figure S17, Supporting Information). This suggests that the charge carriers can be effectively generated in the MOFs microsystems. Furthermore, this photoconductor features a fast response and recovery time at different voltage biases (Figure S18, Supporting Information).

**Figure 3:**
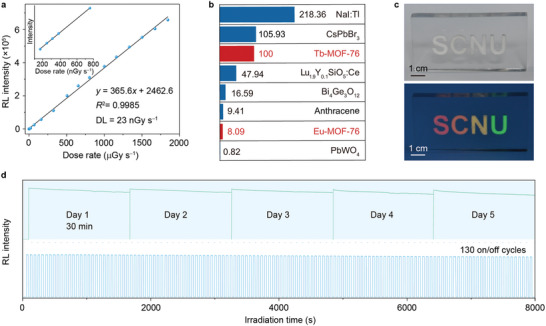
Highly efficient and stable radioluminescence in MOF‐76 microscintillators. a) Recorded radioluminescence intensity of Tb‐MOF‐76 microcrystals as a function of dose rate. The calculated detection limit is 23 nGy s^−1^, with a signal‐to‐noise ratio of 2.85. An enlarged image in the range of 100–800 nGy s^−1^ is shown as an inset. The detection limit is calculated using the 3*σ*/slope method.^[^ ^38^ ^]^ b) Comparison of X‐ray induced luminescence intensity of various scintillator materials at a dose rate of 1845 µGy s^−1^. c) Multicolor visualization using the as‐developed Ln‐MOF scintillating materials upon X‐ray exposure (top, bright‐field imaging; bottom, X‐ray irradiation at a dose rate of 1845 µGy s^−1^). The pits with S, C, N, and U letter shapes were filled with Eu‐MOF‐76, Tb_0.9_Eu_0.1_‐MOF‐76, Tb_0.98_Eu_0.02_‐MOF‐76, and Tb‐MOF‐76 microscintillators, respectively. d) Photostability characterization of Tb‐MOF‐76 microcrystals. Radioluminescence at 546 nm was recorded under continuous X‐ray irradiation for 30 min (top) and 130 on–off cycles of X‐ray exposure with 30 s time interval (bottom).

We next measured the radioluminescence intensities of Tb‐MOFs scintillators and observed a linear correlation with the dose rate of X‐ray irradiation in a wide range (**Figure** **3**a and Figure S19, Supporting Information). The calculated detection limit is 23 nGy s^−1^, ≈240 times lower than the standard dose for X‐ray diagnostics.^[^ ^3^ ^]^ Compared with existing scintillators, the radioluminescence intensity of Tb‐MOF‐76 is comparable to that of high‐efficiency CsPbBr_3_ nanocrystals and much higher than that of Lu_1.9_Y_0.1_SiO_5_:Ce, Bi_4_Ge_3_O_12_, anthracene, and PbWO_4_ scintillators (Figure 3b and Figure S20, Supporting Information). X‐ray flux can be visualized in multicolor due to efficient photoconversion and codoping‐enabled emission color tuning (Figure 3c). The Tb‐MOF‐based scintillator also possesses high photostability, as manifested by a corresponding <10% reduction in radioluminescence intensity during continuous X‐ray irradiation for 30 min or 130 on–off cycles (Figure 3d and Figures S21–S22, Supporting Information).

**Figure 4:**
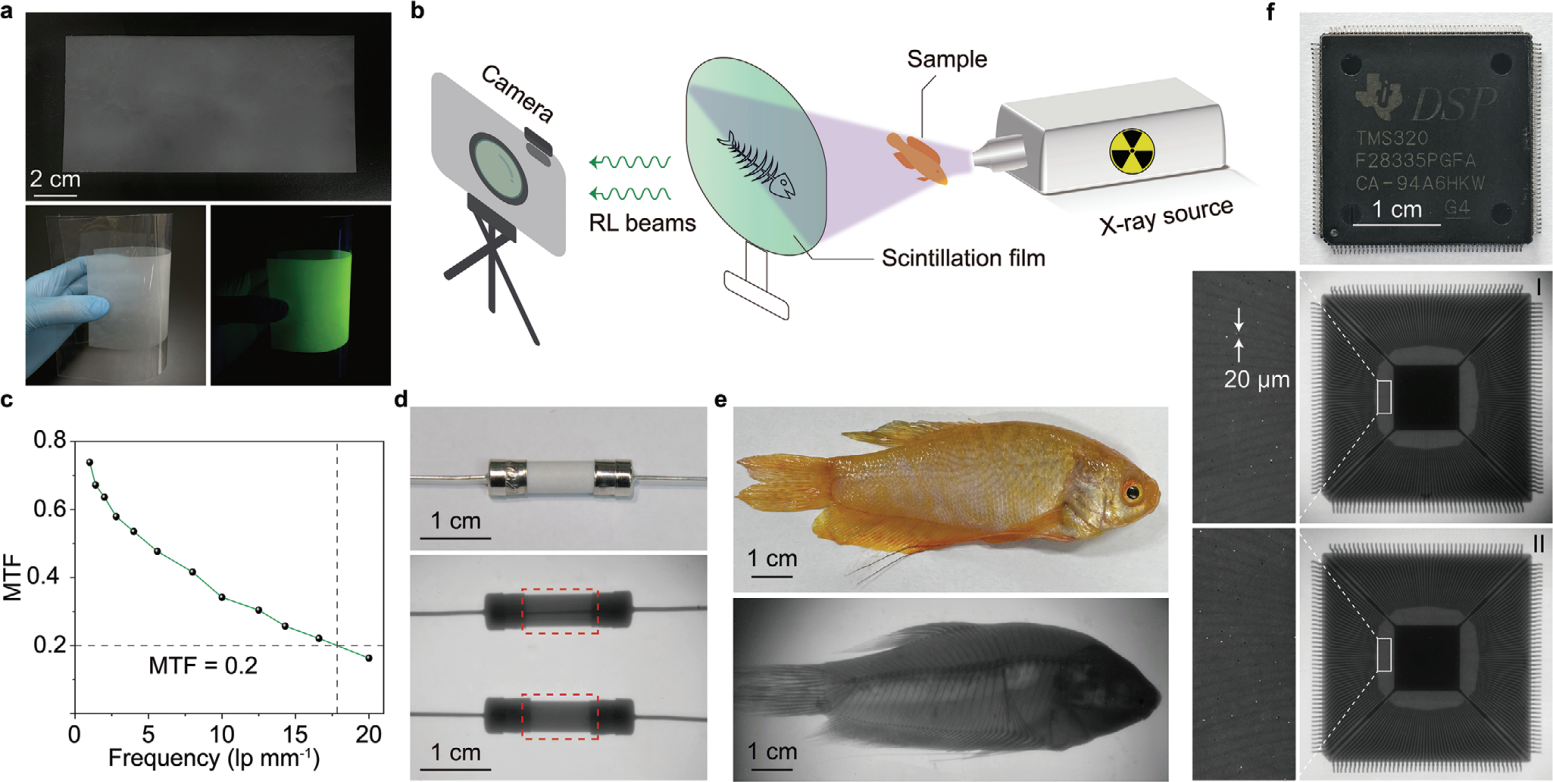
X‐ray radiography using Tb‐MOF‐76 microscintillators. a) Photograph of a 200‐µm‐thick film containing 5 wt% Tb‐MOF‐76 microscintillators (top). The film was fabricated by embedding Tb‐MOF‐76 microcrystals in a polydimethylsiloxane matrix and using poly(vinyl chloride) as the substrate. The large‐area thin‐film shows good flexibility (bottom left) and green emission upon UV irradiation (bottom right). b) Schematic of the X‐ray imaging set‐up. A target sample is placed between the X‐ray source and the scintillation film. Images were recorded using a digital camera. c) MTF values of the Tb‐MOF‐76 film calculated using the line pair pattern method.^[^ ^39^ ^]^ d) Bright‐field image of a ceramic fuse (top) and the corresponding X‐ray images of its inner structure before and after blowing (bottom). e) Bright‐ (top) and dark‐field (bottom) photographs of a fish taken before and after X‐ray exposure. f) Bright‐ (top) and dark‐field (middle and bottom) photographs of an electronic chip. Images I and II were taken with the same Tb‐MOF‐76 film at a time interval of 6 months.

The ability of Tb‐MOF‐76 microcrystals to emit efficient radioluminescence makes them promising for X‐ray radiography. To prove the concept, we prepared a flexible composite film consisting of a transparent polydimethylsiloxane (PDMS) matrix and Tb‐MOF‐76 microcrystals (**Figure** **4**a). Spectroscopic characterization of the scintillation film shows comparable radioluminescence intensity and enhanced photostability compared with pristine Tb‐MOF‐76 microcrystals (Figures S23−S25, Supporting Information). A target object was then placed between the X‐ray source and the Tb‐MOF‐based thin‐film screen, and the image was recorded using a digital camera (Figure 4b). The MOF‐based flexible X‐ray detector exhibits a high spatial image resolution of more than 16.6 line pairs per millimeter (lp mm^−1^) with a modulation transfer function (MTF) of 0.2, which is considerably higher than that achievable by conventional flat‐panel X‐ray detectors (Figure 4c and Figures S26–S27, Supporting Information). Such superior performance could be attributed to the uniform spatial distribution of Tb‐MOF‐76 microcrystals, resulting in less light scattering. The power of X‐ray imaging was further demonstrated by imaging a fish, a ceramic fuse, and an electronic chip (Figure 4d–f). With the scintillator film, a thin wire inside the electronic chip with a diameter of ≈20 µm can be visualized. We did not observe any degradation of spatial resolution in radiology after 6 months of the storage of the scintillation screen under ambient conditions (Figure 4f).

## 3 Conclusion 

In conclusion, we have demonstrated the ability of Ln‐MOF‐based microscintillators to efficiently convert X‐rays into color‐tunable visible light. Benefiting from the heavy‐atom effect and strong electronic coupling at the metal−molecule interface, Tb‐MOF‐76 microscintillators can absorb X‐ray energy, produce abundant electron−hole pairs, and generate high‐density triplet excitons. The combination of triplet excitons with direct trapping of charge carriers cooperatively activates trivalent terbium emitters and enables efficient radioluminescence upon X‐ray irradiation. Compared with conventional inorganic and organic scintillators, Tb‐MOF‐76 microcrystals can be prepared at low temperatures by solution synthesis. They feature excellent photostability, high photoconversion efficiency, and a high degree of spectral modulation, thereby enabling the fabrication of flexible scintillators with excellent visualization and imaging performance. These findings not only demonstrate the potential of MOF‐based microscintillators for applications in optoelectronics and X‐ray imaging but also provide new insights into the design principles for hybrid scintillators.

## 4 Experimental Section

The experimental details are provided in the Supporting Information.

## Conflict of Interest

The authors declare no conflict of interest.

## Supporting information

Supporting Information

## Data Availability

The data that support the findings of this study are available from the corresponding author upon reasonable request.
