# Supplementary material for: High‐Performance X‐Ray Imaging using Lanthanide Metal–Organic Frameworks
Source: Adv Sci (Weinh). 2023 Mar 22;10(15):2207004. doi: 10.1002/advs.202207004 (PMC10214268; doi:10.1002/advs.202207004)
Supplement: Supplementary file 1 — Supporting Information [file ADVS-10-2207004-s001.pdf]

Supporting Information

High-performance X-ray imaging using lanthanide metal-organic frameworks

Xintong Zhang, Haiyi Qiu, Wang Luo, Kaofeng Huang, Ying Chen, Jiacheng Zhang, Bohan Wang, Daoling Peng, Yu Wang, Kezhi Zheng**

Experimental section

Chemicals

1,3,5-benzenetricarboxylate (H_3BTC, 98%), Anthracene (98%), N, N-dimethylformamide (DMF, ≥99.5%), acetone, chloroform, and ethanol (EtOH) (99.9%) were purchased from Aladdin Biochemical Technology. Sylgard® 184 silicone elastomer kit was purchased from Dow Corning. $Ce(NO_3)_3·xH_2O$ (99.9%), $Pr(NO_3)_3·xH_2O$ (99.9%), $Nd(NO_3)_3·xH_2O$ (99.9%), $Sm(NO_3)_3·xH_2O$ (99.9%), $Eu(NO_3)_3·xH_2O$ (99.9%), $Tb(NO_3)_3·xH_2O$ (99.9%), $Dy(NO_3)_3·xH_2O$ (99.9%), $Ho(NO_3)_3·xH_2O$ (99.9%), $Er(NO_3)_3·xH_2O$ (99.9%), $Tm(NO_3)_3·xH_2O$ (99.9%), $TbCl_3·xH_2O$ (99.9%), $Tb(CH_3COO)_3·xH_2O$ (99.9%), Cs_2CO_3 (99.9%), $PbBr_2$ (99.9%), oleylamine (technical grade 70%), oleic acid (technical grade 90%), and 1-octadecene (technical grade 90%) were purchased from Sigma-Aldrich. Crystals of NaI:Tl and $Lu_{1.9}Y_{0.1}SiO_5$:Ce scintillators were purchased from Kinheng Crystal Material Co., Ltd (Shanghai, China). Crystals of $Bi_4Ge_3O_{12}$ and $PbWO_4$ scintillators were purchased from Zhonghelixin Co., Ltd (Chengdu, China). Unless otherwise noted, all chemicals were used without additional treatment.

Synthesis of Tb-MOF-76 microcrystals

Tb-MOF-76 microcrystals were prepared according to the previously reported method with some modifications[1]. In a typical preparation, $Tb(NO_3)_3·xH_2O$ and H_3BTC with a molar ratio of 3:1 were dissolved in 8 mL DMF and 2 mL H_2O by ultrasonication for 5 min at room temperature. The mixture was then sealed and heated at 70 °C in a water bath for 12 h. Finally, the as-synthesized Tb-MOF-76 microcrystals were rinsed with DMF and ethanol for several times and dried in the oven at 70 °C for 24 h. To investigate the effect of experimental parameters on the structure, size, shape, and light output of final samples, a series of Tb-MOF-76 materials were synthesized at different reaction temperature (50 °C, 60 °C, 70 °C, 80 °C, and 90 °C) with different terbium precursors ($Tb(NO_3)_3·xH_2O$, $TbCl_3·xH_2O$, and $Tb(CH_3COO)_3·xH_2O$) and reactant ratios (Tb/BTC = 1/2, 1, 2, 3, 4, and 5).

Synthesis of Tb_xEu_{1-x}-MOF-76 (x = 0, 0.9, and 0.98) microcrystals

The synthetic procedure for Tb_xEu_{1-x}-MOF-76 microcrystals was identical to that of Tb-MOF-76 except for the use of different reactant ratios between Tb and Eu.

Preparation of purified Anthracene crystals

Anthracene crystals were obtained using slow solvent evaporation crystallization techniques[2]. A saturated solution of anthracene in chloroform solvent was prepared for slow evaporation. This procedure was repeated several times for obtaining highly purified anthracene crystals.

Preparation of $CsPbBr_3$ perovskite nanocrystals

$CsPbBr_3$ perovskite nanocrystals were prepared by a hot-injection procedure according to Ref. S3.

Preparation of Tb-MOF-76-Polydimethylsiloxane composite film

In our experiment, Sylgard® 184 silicone elastomer base was premixed with the curing agent (10:1 by mass). The Tb-MOF-76 microcrystals (0.2 g) after grinding in agate mortar was embedded into the resultant solution (4g) and stirred vigorously. The mixture was evenly coated onto the poly(Vinyl chloride) PVC film by metal applicator (200 μm). After degassed for 20 min, the coating materials were heated to 70°C and kept for 8 h before cooling down to room temperature. The resulting Tb-MOF-76-polydimethylsiloxane composite film is attached conformably to the PVC substrate.

Preparation of Tb-MOF-76-based photodetector

To fabricate the photodetector, glass substrates (30 mm × 10 mm) on which two separate indium tin oxide (ITO) electrodes were deposited with a gap of 100 μm were purchased from Guluo Glass Co., Ltd (Luoyang, China). These substrates were first cleaned by sonication in acetone and ethanol separately and dried with flowing nitrogen. The Tb-MOF-76 microcrystals (0.1 g) were dispersed into 1 mL of ethanol at room temperature by sonication, then the prepared solution was spin-coated onto the glass-substrate at 600 r.p.m. for 20 s and subsequently annealed at 90°C for 5 min. This procedure was repeated several times to fabricate a film between ITO electrodes with a thickness of few hundred microns.

Characterization

Scanning electron microscopy (SEM) and energy dispersive X-ray spectroscopy (EDX) characterization were performed using a field emission scanning electron microscope (ZEISS Gemini 500). X-ray diffraction (XRD) patterns were obtained using an X-ray polycrystalline diffractometer (BRUKER D8 ADVANCE) over the angular range of 10–60° at room temperature. X-ray photoelectron spectroscopy (XPS) analysis was performed using an X-ray photoelectron spectrometer (AXIS SUPRA). Radioluminescence spectra were recorded by an Omni-λ 300i fluorescence spectrophotometer (Zolix Instruments Co., Ltd, Beijing, China) equipped with a miniature X-ray source (TUB00154-9I-W06, Moxtek, Inc. USA). Steady-state photoluminescence excitation and emission spectra and time-dependent photoluminescence spectra were recorded using an FLS1000 fluorescence spectrograph (Edinburgh Instruments Ltd, UK) equipped with a xenon arc lamp or microsecond flash. Photographs of X-ray-induced luminescence and X-ray imaging were acquired with a digital camera (Canon EOS R5 with RF100mm F2.8 L MACRO IS USM; shutter speed: 15 s; aperture: 2.8; ISO: 1600). The voltage/current of the X-ray tube was set to 40 kV/200 μA for X-ray imaging. For X-ray photon-to-current measurement, a miniature X-ray tube was used as excitation source. In our measurement, the distance between the X-ray source and the MOF-based photodetector was about 10 cm. The current-voltage measurement was performed using a Keysight 2912 source meter. All experiments were carried out at ambient conditions.

The X-ray absorption spectra were calculated from a web program called XCOM, which provides a comprehensive database that can be used to calculate photon cross sections for scattering, photoelectric absorption and pair production, as well as total attenuation coefficients, for any element, compound or mixture ($Z \leqslant 100$), at energies from 1 keV to 100 GeV (cited from M. J. Berger, J.H. Hubbell, S.M. Seltzer, J. Chang, J.S. Coursey, R. Sukumar, D.S. Zucker, K. Olsen, XCOM: Photon Cross Sections Database, (NIST, 2013); https://www.nist.gov/pml/xcom-photon-cross-sections-database).

The modulation transfer function (MTF), which is defined as the transfer capability of the input signal modulation of spatial frequency, serves as an important index to evaluate the spatial resolution performance of our X-ray imaging system. When the MTF value decreases to 0.2, the spatial resolution can be determined from the corresponding spatial frequency.

Density functional theory calculation

To optimize the geometry structure and analyze the harmonic frequency of an isolated 1,3,5-benzenetricarboxylate (H_3BTC) molecule, density functional theory (DFT) calculations were performed using Gaussian 16 (A.03) program[4] in which M06-2X functional in combination with 6-311G** basis set were used[5, 6]. The optimized molecular structure shows no imaginary frequency and features a stable wavefunction, suggesting that the genuine ground state of H_3BTC molecules has been reached. Time-dependent density functional theory (TD-DFT) method was employed to calculate the S_1 and T_1 vertical absorption energies of H_3BTC molecules. Note that for excited-state calculation, we used the same functional as ground-state calculation, while the def2-TZVP basis set was used.

Given the crystalline nature of MOFs, we investigated the ground-state electronic structures of Tb-MOF-76 and Eu-MOF-76 through DFT calculations with periodic boundary conditions. The quantum calculations were conducted using the Vienna ab initio package with the projector augmented wave method[7,8]. The Perdew-Burke-Ernzerh generalized gradient

approximation was employed to describe the exchange-correlation interactions between electrons[9]. Note that the screened-exchange hybrid density functional HSE06 with 12% Hartree-Fock exchange interaction was used to probe the split of Tb's 4f orbitals[10]. The kinetic energy cut-off, energy convergence criterion, and maximum residual force were set to 500 eV, 1×10^{-6} eV, and 0.01 eV/Å, respectively.

Supplementary References

1. Yao, Y. et al. Heteroepitaxial Growth of Multiblock Ln-MOF Microrods for Photonic Barcodes. *Angew. Chem. Int. Ed.* **58**, 13803–13807 (2019).
2. Madhurambal, G. & Srinivasan, P. A. Growth of high quality anthracene crystals by a simple solution technique. *Cryst. Res. Technol.* **41**, 231–235 (2006).
3. Chen, Q. et al. All-inorganic perovskite nanocrystal scintillators. *Nature* **561**, 88–93 (2018).
4. Frisch, M. J. et al. Gaussian 16, Revision A.03, Gaussian, Inc., Wallingford CT (2016).
5. Zhao, Y. & Truhlar, D. G. *Theor. Chem. Acc.* **120**, 215 (2008).
6. Hehre, W. J. Random, L. Schleyer, P. v. R. & Pople, J. A. Ab Initio molecular orbital theory. *Wiley: New York* (1986).
7. Kresse, G. & Furthmüller, J. Efficiency of ab-initio total energy calculations for metals and semiconductors using a plane-wave basis set. *Comput. Mater. Sci.* **6**, 15–50 (1996).
8. Blöchl, P. E. Projector augmented-wave method. *Phys. Rev. B* **50**, 17953–17979 (1994).
9. Perdew, J. P. Burke, K. & Ernzerhof, M. Generalized gradient approximation made simple. *Phys. Rev. Lett.* **77**, 3865–3868 (1996).
10. Heyd, J. Scuseria, G. E. & Ernzerhof, M. Hybrid functionals based on a screened Coulomb potential. *J. Chem. Phys.* **118**, 8207–8215 (2003); correction **124**, 219906 (2006).

Table S1. Luminescence lifetime of lanthanide-based MOF-76 microcrystals.[a]

Compound	Wavelength (nm)	Fluorescence					
		τ_1 (ms)	A_1	τ_2 (ms)	A_2	τ (ms)	R^2
Eu-MOF-76	616	0.71	5.29	0.71	5.25	0.71	0.99965
$Tb_{0.9}Eu_{0.1}$-MOF-76	616	0.83	61.68	0.83	60.33	0.83	0.99944
	546	0.14	60.43	0.44	42.84	0.35	0.99889
$Tb_{0.98}Eu_{0.02}$-MOF-76	616	1.15	63.41	1.15	62.04	1.15	0.99822
	546	0.24	21.77	0.95	81.61	0.91	0.99956
Tb-MOF-76	546	0.12	1.77	1.40	101.30	1.40	0.99964

[a] Determined from the fitting function of $I(t) = A_1 \exp(-\frac{t}{\tau_1}) + A_2 \exp(-\frac{t}{\tau_2})$ according to luminescence decay curves, where A_i is the pre-exponential factor for lifetime τ_i. The average lifetime of luminescence is calculated by $\tau = \sum A_i\tau_i^2 / \sum A_i\tau_i$.

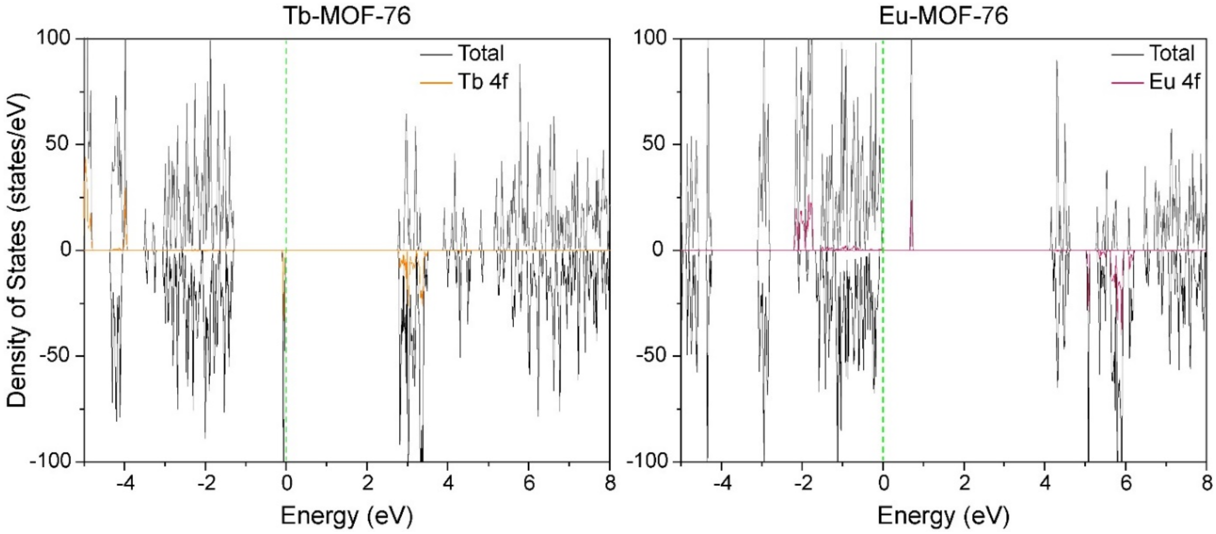

Figure S1. Calculated density of states (DOS) of Tb-MOF-76 and Eu-MOF-76 microcrystals. Green dotted lines indicate the position of the Fermi level.

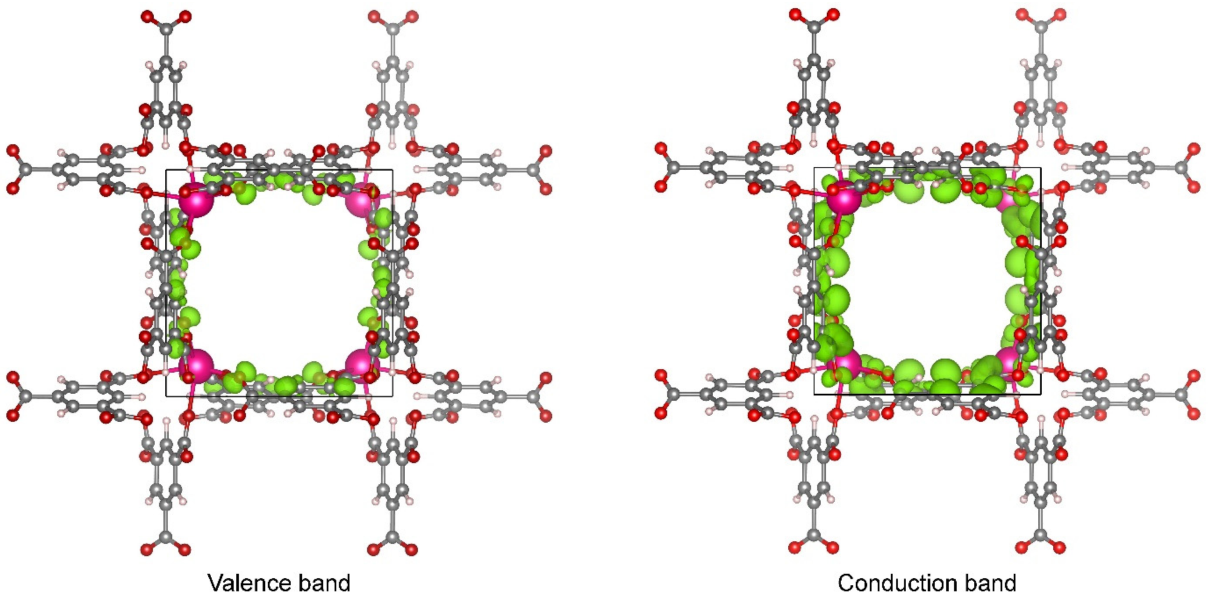

Figure S2. Calculated partial charge density (in green) related to the valence and conduction band edges of Eu-MOF-76 microcrystals. Note that magenta, gray, red, and pink balls represent Eu, C, O, and H atoms, respectively.

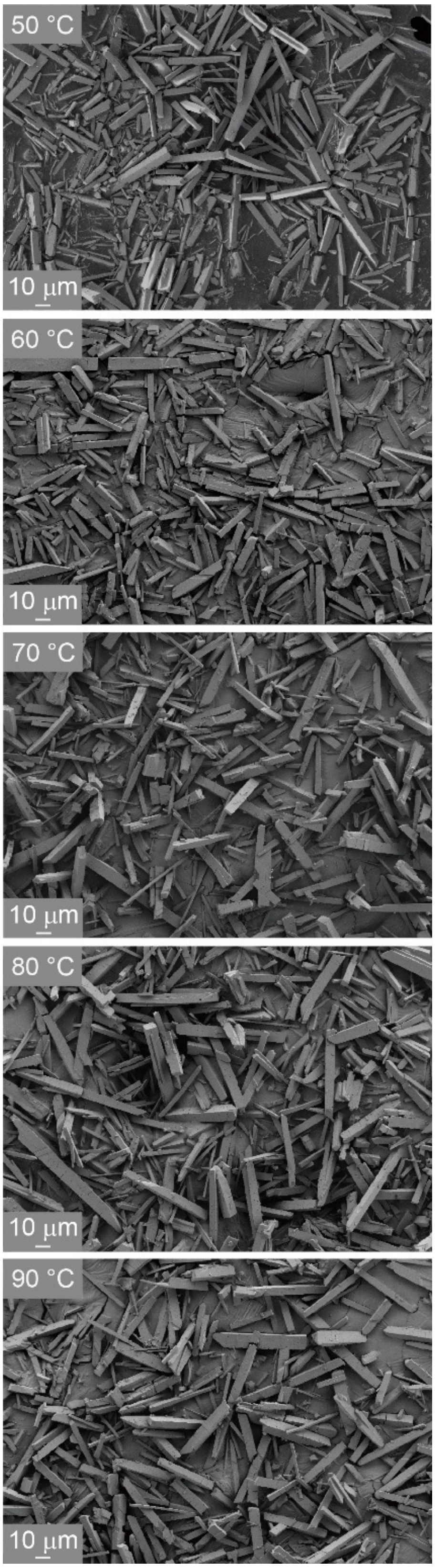

Figure S3. SEM images of Tb-MOF-76 microcrystals synthesized at different temperature (50 °C, 60 °C, 70 °C, 80 °C, and 90 °C) with a Tb/BTC ratio of 3. Terbium nitrate was used as terbium precursor.

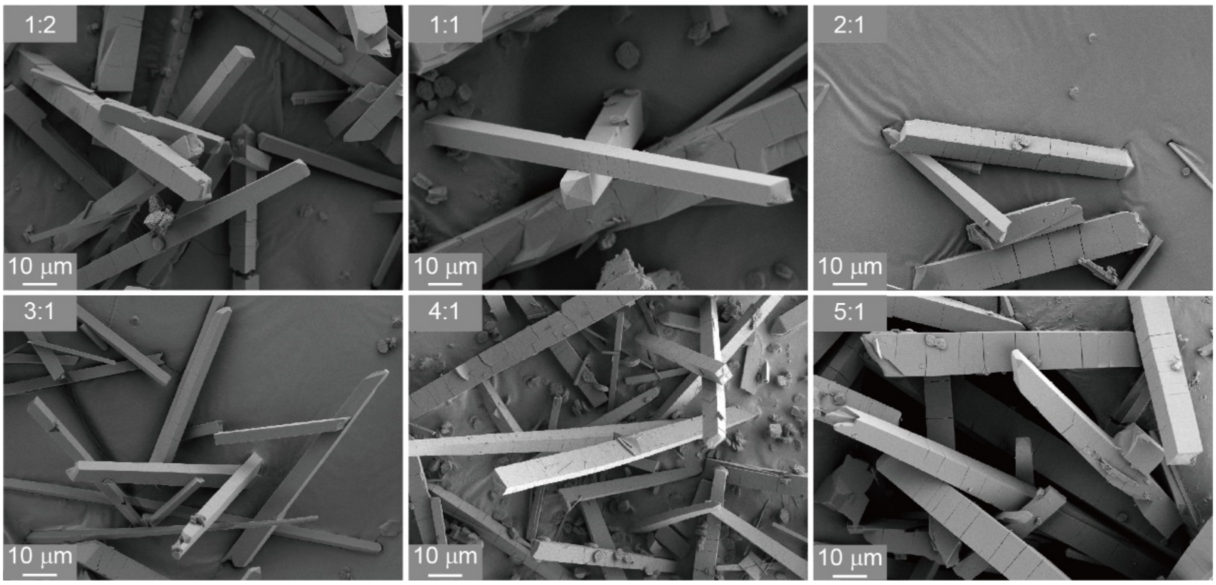

Figure S4. SEM images of the Tb-MOF-76 microcrystals synthesized at 70 °C with different Tb/BTC ratios (1/2, 1, 2, 3, 4, and 5). Terbium nitrate was used as terbium precursor.

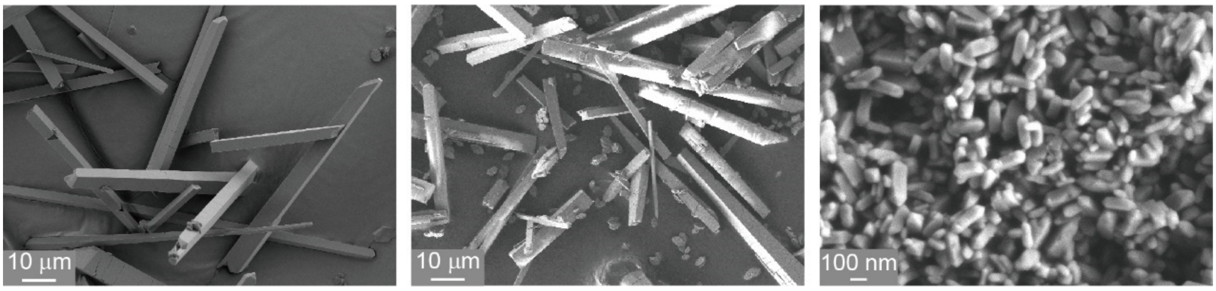

Figure S5. SEM images of Tb-MOF-76 crystals synthesized at 70 °C with a Tb/BTC ratio of 3. Terbium nitrate (left), terbium chloride (middle), and terbium acetate (right) were used as terbium precursor, respectively.

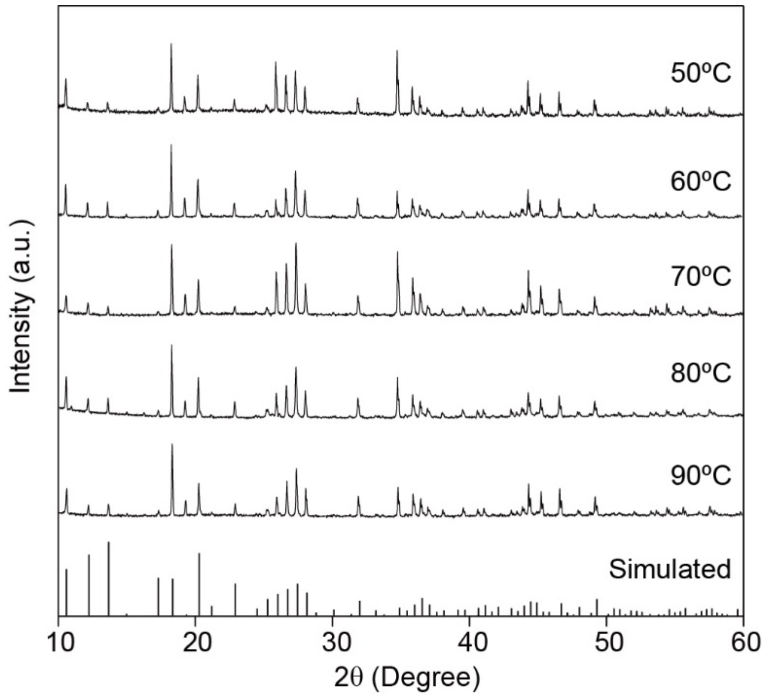

Figure S6. X-ray diffraction patterns of Tb-MOF-76 microcrystals synthesized at different temperatures (50 °C, 60 °C, 70 °C, 80 °C, and 90 °C) with a Tb/BTC ratio of 3 using terbium nitrate as terbium precursor. All peaks are indexed with the simulated structure of MOF-76.

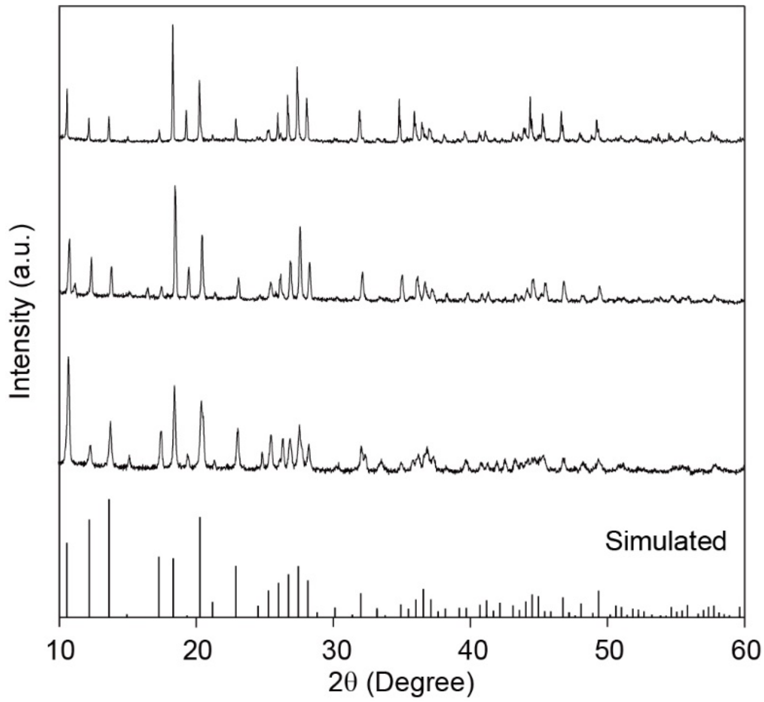

Figure S7. X-ray diffraction patterns of Tb-MOF-76 crystals synthesized at 70 °C with a Tb/BTC ratio of 3. Terbium nitrate (top), terbium chloride (middle), and terbium acetate (bottom) were used as terbium precursor, respectively. All peaks are indexed with the simulated structure of MOF-76.

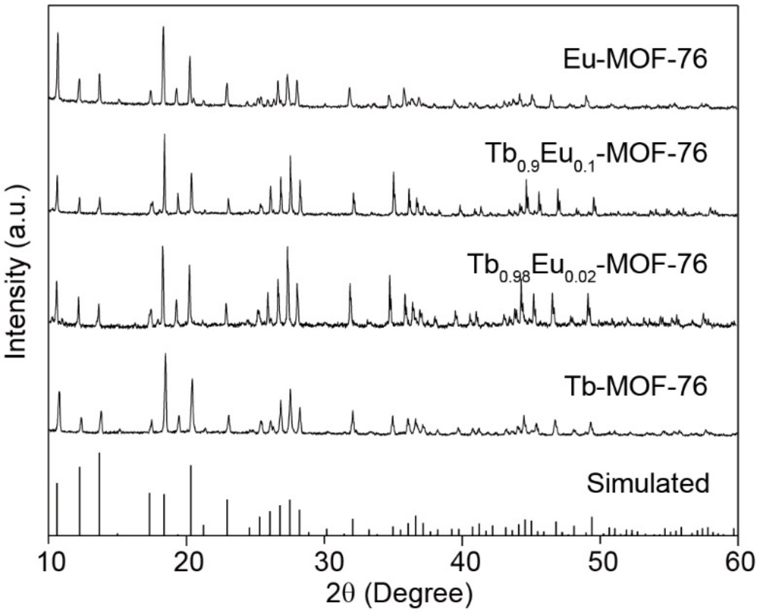

Figure S8. X-ray diffraction patterns of four MOF-76 microcrystals synthesized at 70 °C with different lanthanide dopants. All peaks are indexed with the simulated structure of MOF-76.

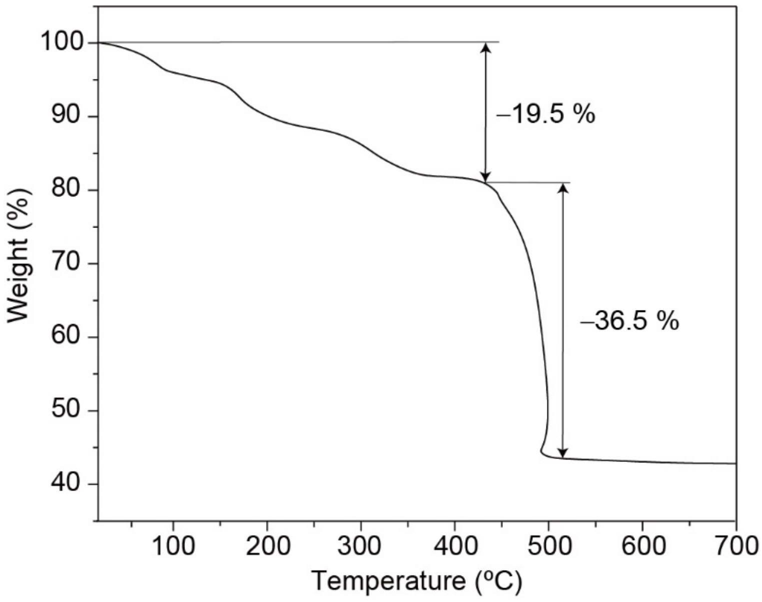

Figure S9. Thermogravimetry analysis (TGA) curve of the as-synthesized Tb-MOF-76 microcrystals. A weight loss of 19.5 % was observed when heated to 440°C, which corresponds to the desorption of water and DMF molecules. Rapid weight loss above 440°C could be attributed to the collapse of MOF structure.

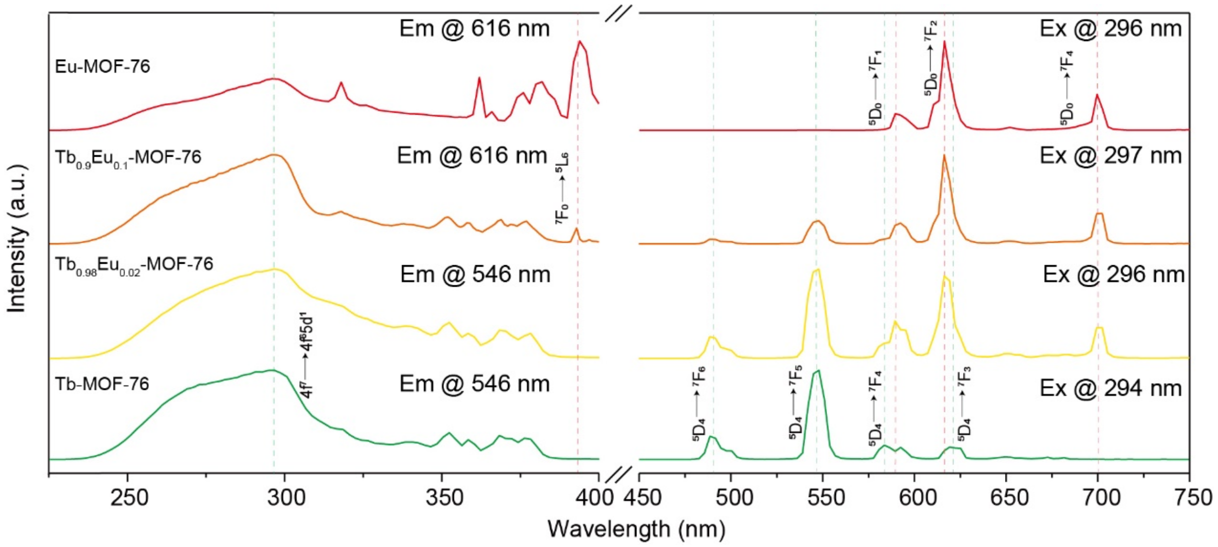

Figure S10. Photoluminescence excitation (left) and emission (right) spectra of four MOF-76 microcrystals with different lanthanide dopants.

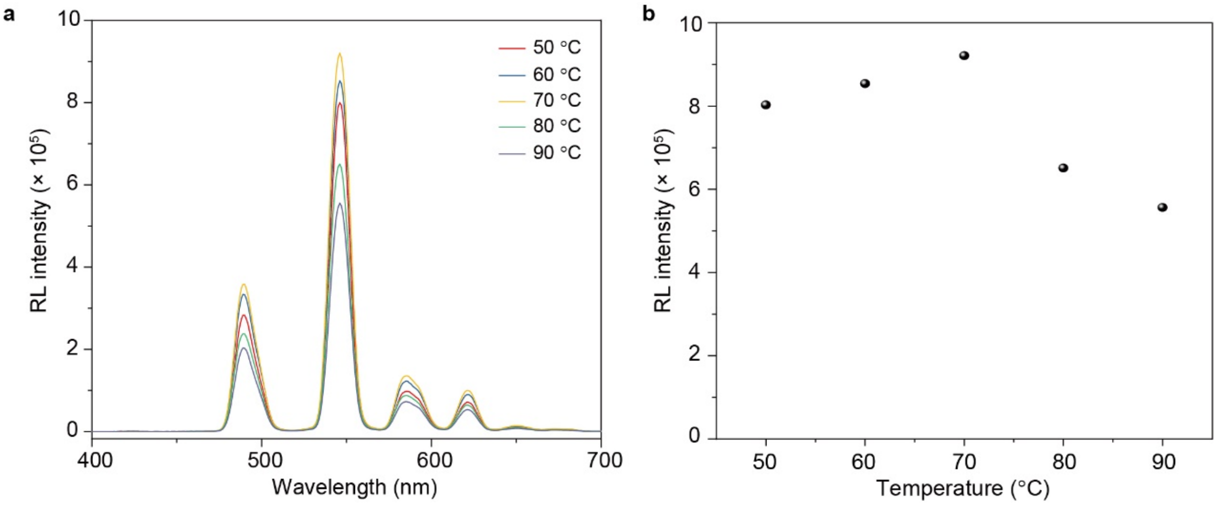

Figure S11. a, Radioluminescence spectra of Tb-MOF-76 microcrystals synthesized at different temperatures (50 °C, 60 °C, 70 °C, 80 °C, and 90 °C) with a Tb/BTC ratio of 3 (dose rate, 1845 μGy s^{-1}). **b,** The corresponding intensity dependence of 546-nm emission on the synthetic temperature.

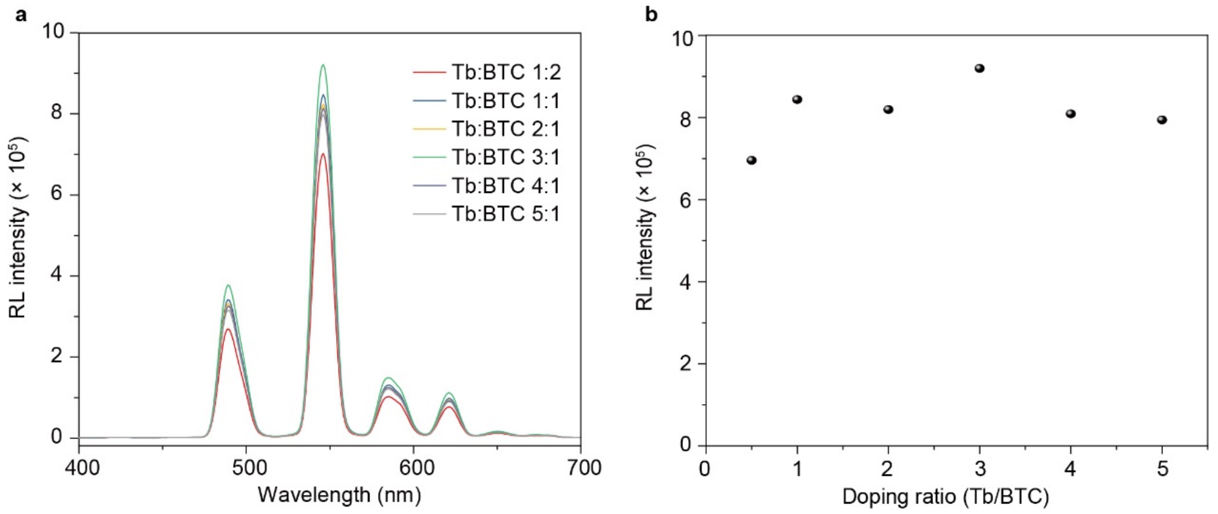

Figure S12. a, Radioluminescence spectra of Tb-MOF-76 microcrystals synthesized at 70 °C with different Tb/BTC ratios (1/2, 1, 2, 3, 4, and 5) (dose rate, 1845 μGy s^{-1}). **b,** The corresponding intensity dependence of 546-nm emission on the ratio of Tb to BTC.

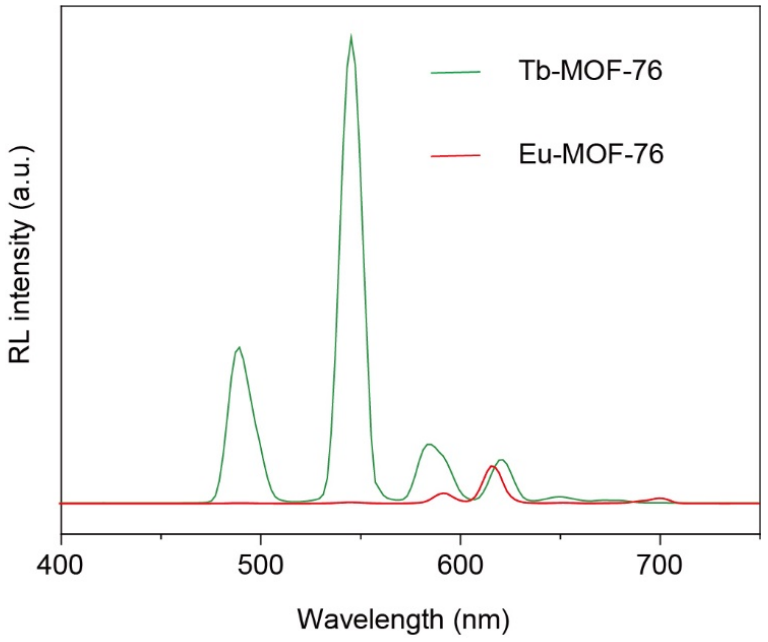

Figure S13. Radioluminescence spectra of Tb-MOF-76 and Eu-MOF-76 microcrystals, recorded at an X-ray dose rate of 1845 μGy s^{-1}.

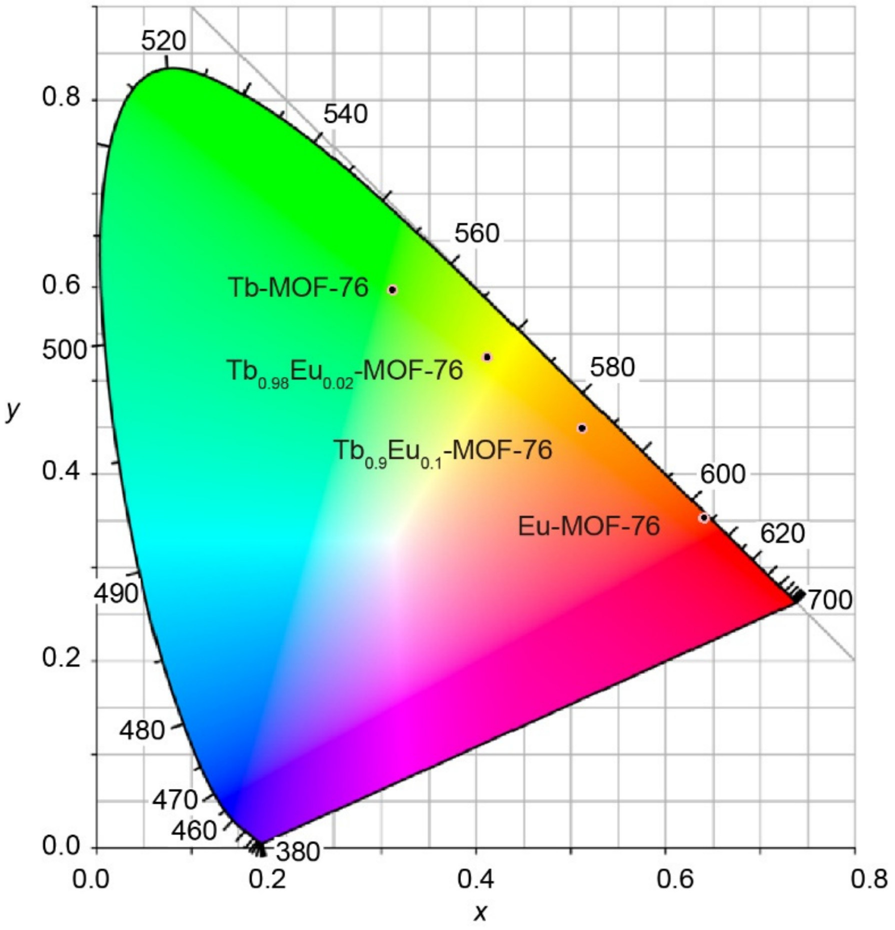

Figure S14. CIE (Commission Internationale de l'Eclairage) chromaticity coordinates of X-ray-induced visible emissions of Tb-MOF-76, $Tb_{0.98}Eu_{0.02}$-MOF-76, $Tb_{0.9}Eu_{0.1}$-MOF-76, and Eu-MOF-76 microcrystals.

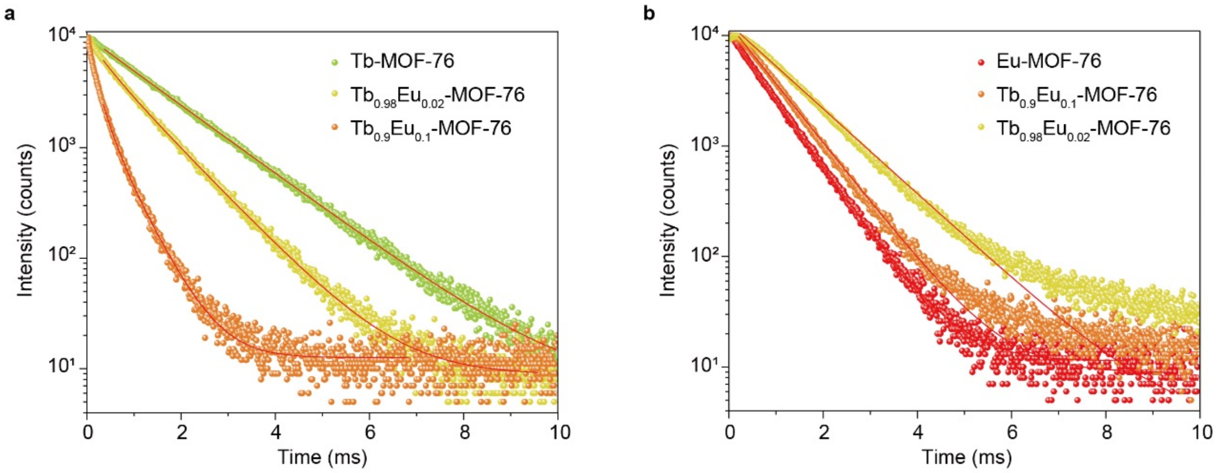

c

	$\tau_{546\ nm}$ (ms)	$\tau_{616\ nm}$ (ms)
Tb-MOF-76	1.4	–
$Tb_{0.98}Eu_{0.02}$-MOF-76	0.91	1.15
$Tb_{0.9}Eu_{0.1}$-MOF-76	0.35	0.83
Eu-MOF-76	–	0.71

Figure S15. Lifetime profiles of **(a)** Tb at 546 nm and **(b)** Eu at 616 nm in MOF-76 microcrystals (λ_{ex} = 296 nm). Note that the average lifetime was calculated according to $\tau = \sum A_i\tau_i^2 / \sum A_i\tau_i$. **(c)** Compiled lifetime of lanthanide emitters.

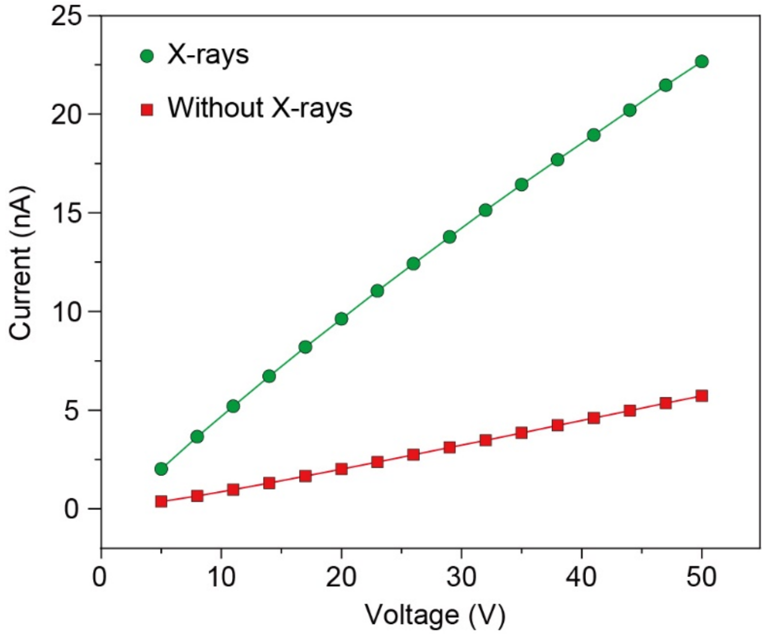

Figure S16. *I-V* characteristics of the as-prepared photodetector, measured with and without X-ray illumination (dose rate, 1845 μGy s^{-1}).

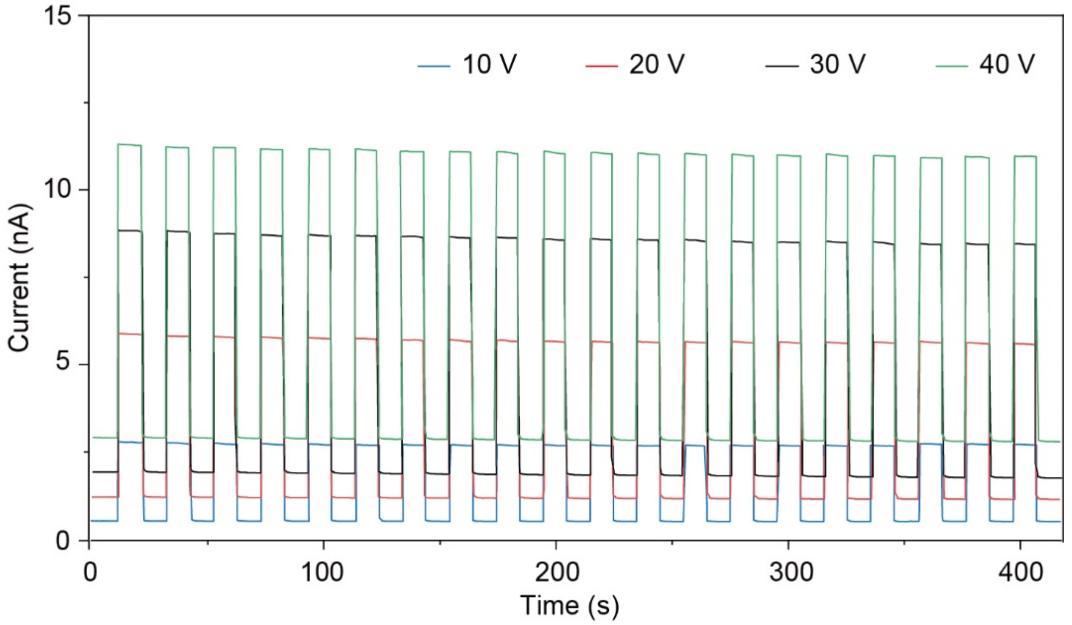

Figure S17. Current dynamic responses of the homemade photodetector under various bias voltages (from 40 to 10 V) upon pulsed X-ray excitation with a time interval of 10 s (dose rate, 1845 μGy s^{-1}).

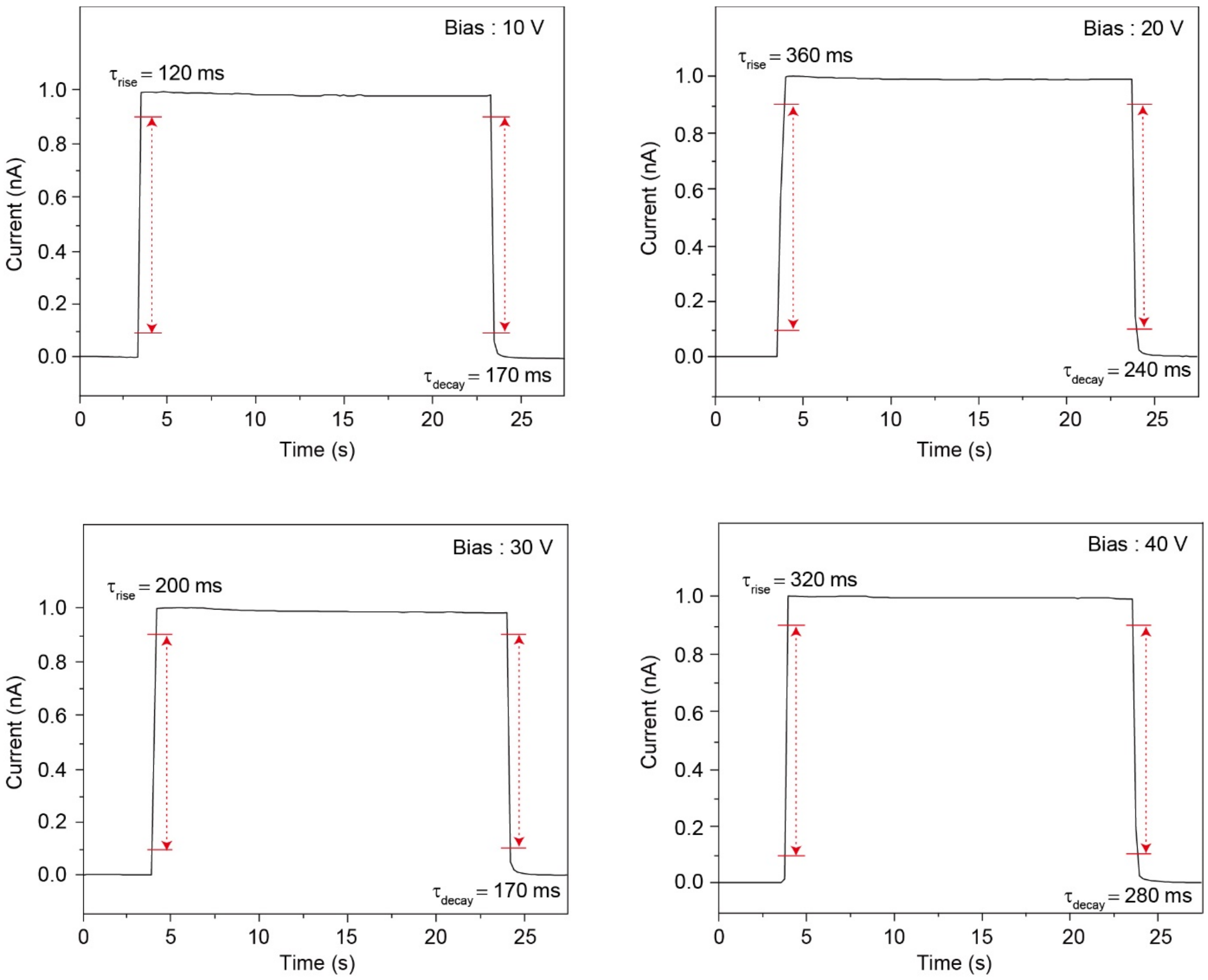

Figure S18. Time-resolved photocurrent curve of Tb-MOF-76-based detector, showing the corresponding rise and decay time under X-ray illumination (bias voltage, 10–40 V; dose rate, 1845 μGy s^{-1}).

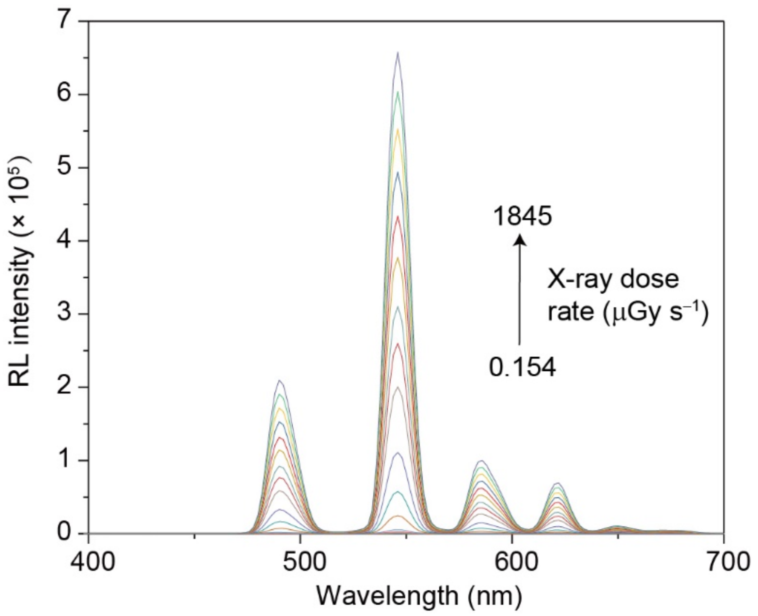

Figure S19. Radioluminescence spectra of Tb-MOF-76 microcrystals, recorded at different X-ray dose rates (from 0.154 to 1845 μGy s^{-1}).

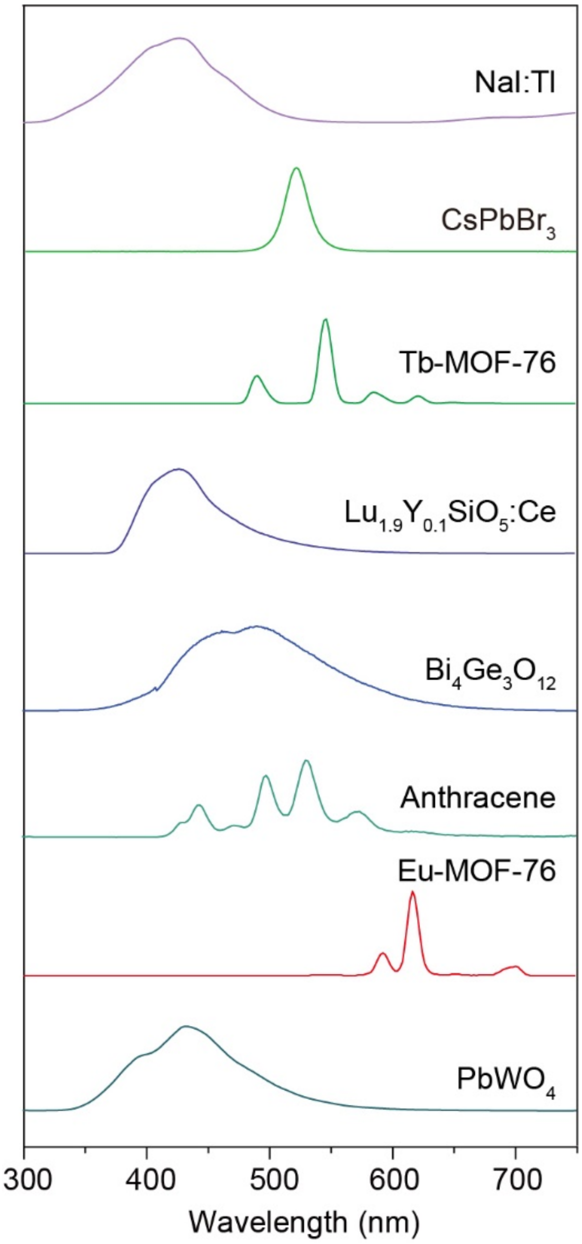

Figure S20. X-ray-excited luminescence spectra of Tb-MOF-76 and Eu-MOF-76 microcrystals, NaI: Tl crystal, $CsPbBr_3$ nanocrystal, $Lu_{1.9}Y_{0.1}SiO_5$: Ce crystal, $Bi_4Ge_3O_{12}$ crystal, anthracene crystal, and $PbWO_4$ crystal. Note that each curve was normalized to its maximum intensity.

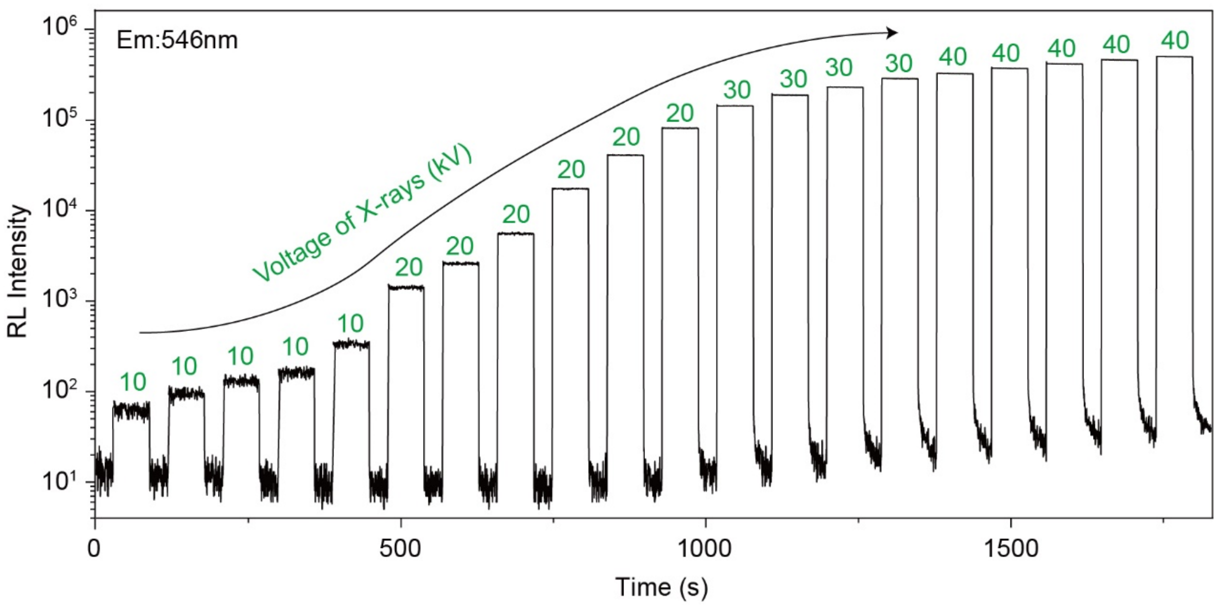

Figure S21. Kinetic measurement of emission intensity at 546 nm of Tb-MOF-76 microcrystals, recorded at different X-ray dose rates (from 0.154 to 1845 μGy s^{-1}).

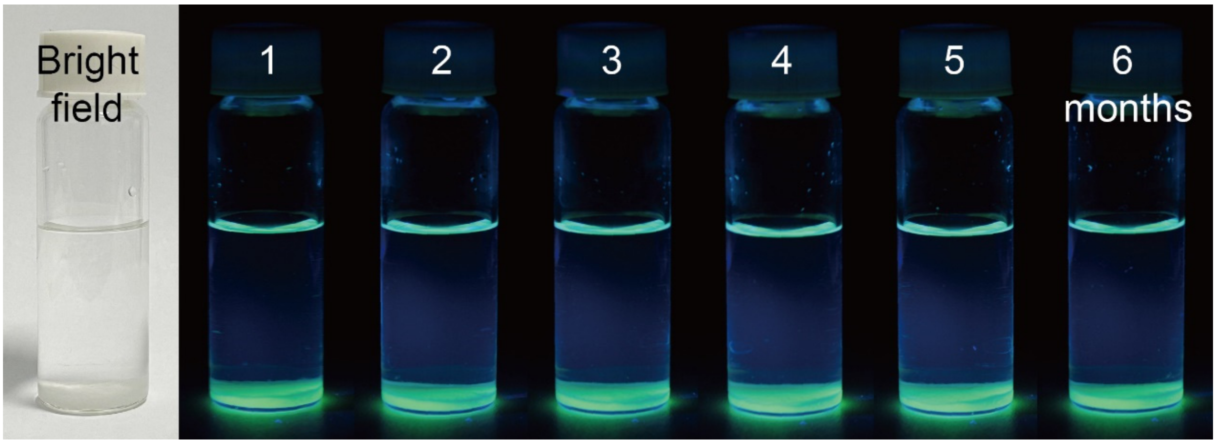

Figure S22. Bright field image and X-ray-excited luminescence photographs of Tb-MOF-76 microcrystals in water–DMF solvent taken at different time intervals (dose rate, 1845 μGy s^{-1}).

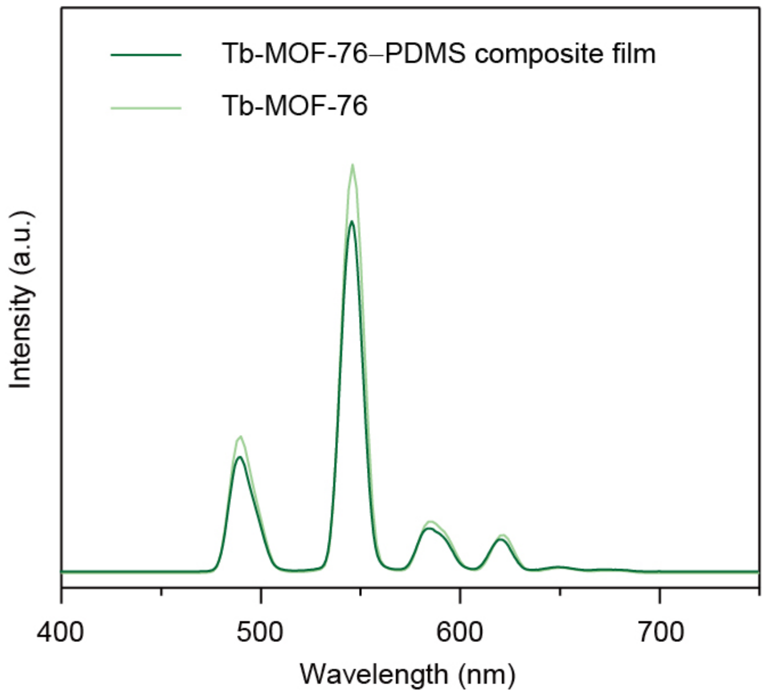

Figure S23. Radioluminescence spectra of Tb-MOF-76-PDMS composite film (top) and pristine Tb-MOF-76 microcrystals (bottom), recorded at an X-ray dose rate of 1845 μGy s^{-1}.

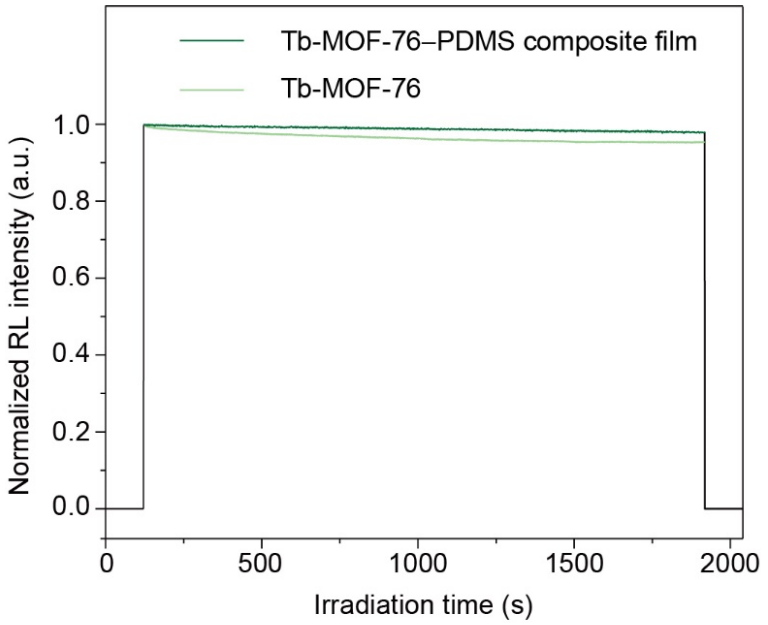

Figure S24. Photostability characterization of Tb-MOF-76-PDMS composite film and pristine Tb-MOF-76 microcrystals at 546 nm under continuous X-ray irradiation at a dose rate of 1845 μGy s^{-1}.

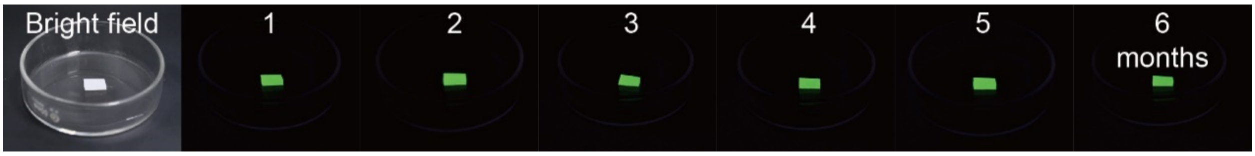

Figure S25. Bright field image and X-ray-excited luminescence photographs of Tb-MOF-76-PDMS composite film taken at different time intervals (dose rate, 1845 μGy s^{-1}). Note that the composite film was stored under ambient conditions.

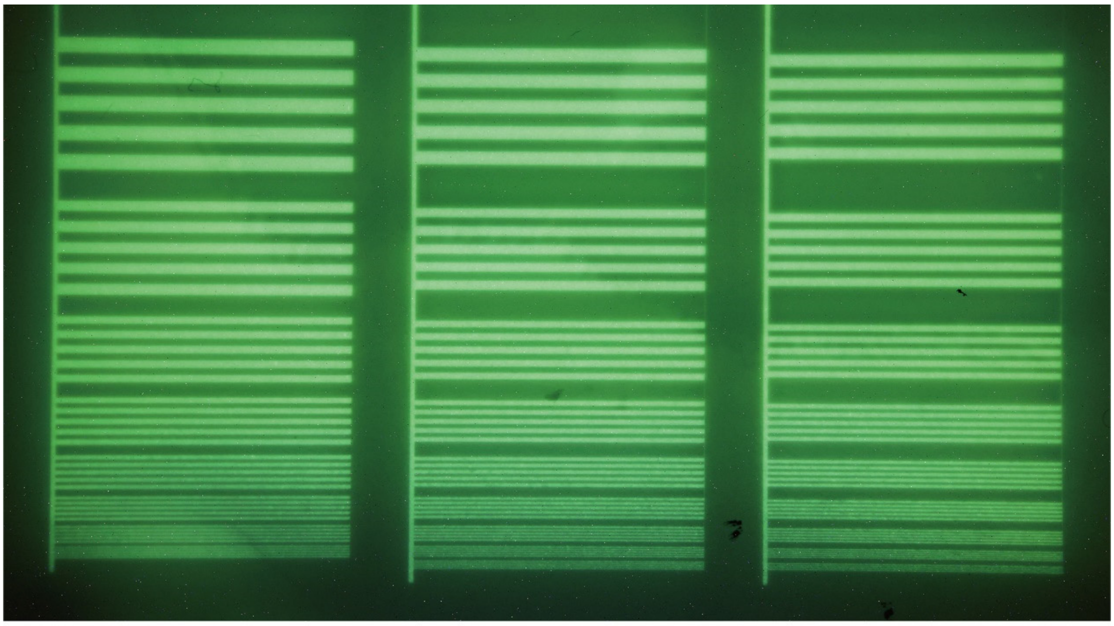

1.0	1.12	1.25
1.4	1.6	1.8
2.0	2.24	2.5
2.8	3.15	3.55
4.0	4.5	5.0
5.6	6.3	7.1
8.0	8.9	10.0
11.1	12.5	14.3
	16.6	20.0

Figure S26. Spatial resolution of digital X-ray imaging measured by a line pair card (Typ 18d). The corresponding resolution value (1.0–20 lp mm^{-1}) of the line pair card is listed as well.

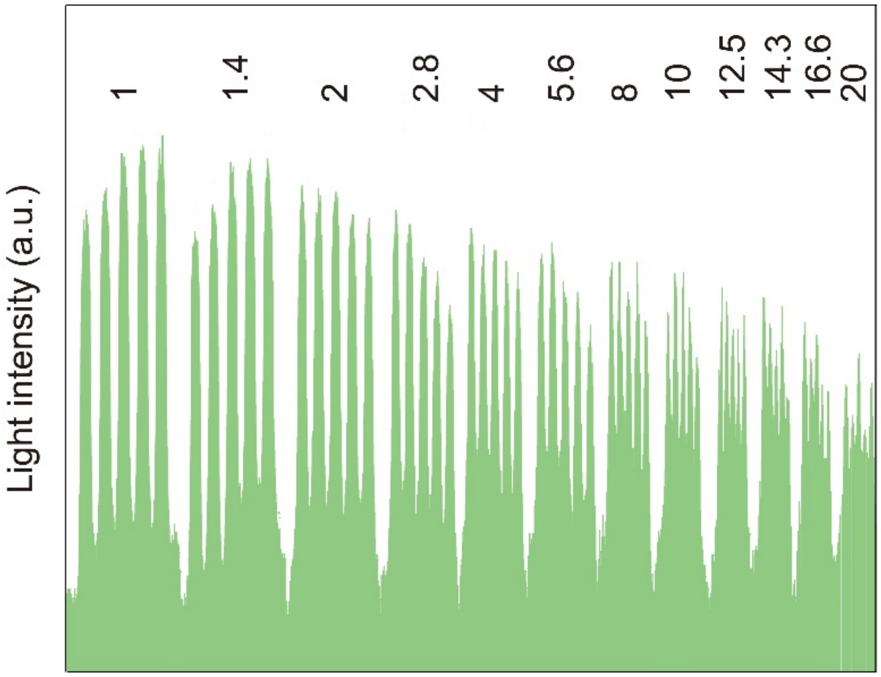

Figure S27. Light intensity function of X-ray imaging of the line pair card.
